# Mapping oral health related quality of life to generic health state values

**DOI:** 10.1186/1472-6963-6-96

**Published:** 2006-08-07

**Authors:** David S Brennan, A John Spencer

**Affiliations:** 1 Australian Research Centre for Population Oral Health, School of Dentistry, Faculty of Health Sciences, The University of Adelaide, South Australia

## Abstract

**Background:**

A summary utility index is useful for deriving quality-adjusted life years (QALY) for cost analyses or disability weights for burden of disease studies. However, many quality of life instruments provide descriptive profiles rather than a single utility index. Transforming quality of life instruments to a utility index could extend the use of quality of life instruments to costs analyses and burden of disease studies. The aims of the study were to map a specific oral health measure, the Oral Health Impact Profile to a generic health state measure, the EuroQol, in order to enable the estimation of health state values based on OHIP data.

**Methods:**

Data were collected from patients treated by a random sample of South Australian dentists in 2001–02 using mailed self-complete questionnaires. Dentists recorded the diagnosis of dental conditions and provided patients with self-complete questionnaires to record the nature, severity and duration of symptoms using the EuroQol (EQ-5D) and 14-item version of the Oral Health Impact Profile (OHIP-14) instruments. Data were available from 375 patients (response rate = 72%). A random two-thirds sample of patients was used in tobit regressions of EQ-5D health state values estimated using OHIP-14 in a model with categories of OHIP responses as indicator variables and in a model with OHIP responses as continuous variables. Age and sex were included as covariates in both models. The remaining one-third sample of patients was used to test the models.

**Results:**

The OHIP item 'painful aching in mouth' was significantly related to health state values in both models while 'life less satisfying' was also significant in the continuous model. Mean forecast errors relative to the mean observed health state value were higher when fitted to the categorical model (17.4%) compared to the continuous model (15.2%) (P < 0.05).

**Conclusion:**

The findings enable health state values to be derived from OHIP-14 scores for populations where utility has not or cannot be measured directly.

## Background

Oral health related quality of life measures have been developed because objective clinical measures of disease provided little insight into the impact of oral disorders on daily living and quality of life [1]. The value of oral health related quality of life measures in the description of the experience of disease and treatment could be enhanced further through the development of a summary utility index that could measure health state values on a scale from zero to one where zero represents worst health and one best health [2]. Transforming health related quality of life instruments to a summary utility index is useful for deriving quality-adjusted life years (QALY) for cost analyses [3], or disability weights for burden of disease studies [4].

The Oral Health Impact Profile (OHIP) is a disease-specific measure of people's perceptions of the social impact of oral disorders on their well-being [5]. OHIP contains 49 questions that capture seven conceptually formulated dimensions based on Locker's theoretical model of oral health [6], and the OHIP-14 was developed as a shorter version of the OHIP for settings where the full battery is inappropriate [7]. While the OHIP is widely used as a measure of oral health related quality of life it does not provide an index of health state value.

The EuroQol was developed as a standardised generic (non-disease-specific) instrument for describing and valuing health-related quality of life [8]. The EuroQol is intended to complement other forms of quality of life measures and it was purposefully developed to generate a generic index of health that places health states on scale from zero (worst health) to one (best health). The EuroQol is widely used internationally and reported to have adequate construct and convergent validity, but is highly skewed and has relatively poor sensitivity especially in relation to disease-based outcomes research [9].

The EuroQol is an index measure that provides a single number that represents an individuals' health status and preference value for that health state [10]. The EuroQol has been used in population surveys such as in the U.K. to establish population norms for the instrument [11], and has been linked to the development of disability weights that have application in burden of disease studies based on disability-adjusted life years [12]. Disability-adjusted life years were developed in conjunction with the World Bank and have been used for priority setting in health policy [13], for example, by the World Health Organization [14]. In Australia the Australian Burden of Disease and Injury Study used the EuroQol to estimate disability weights for a range of disease categories for which there were no published weights [15].

The aims of the study were to map a specific oral health measure, the Oral Health Impact Profile to a generic health state measure, the EuroQol, in order to enable the estimation of health state values based on OHIP data.

## Methods

### Ethical review

The research project was reviewed and approved by the Human Research Ethics Committee of the University of Adelaide.

### Design

The Burden of Oral Disease Study was conducted as a cross-sectional study using a mailed survey approach. Dentists were provided with a practitioner logbook in the dentist questionnaire to record for the first 1 to 5 consecutive adult patients (depending on study group assignment of dentist) of a random clinical day the diagnosis of the oral disease or condition treated and treatment they performed. At the conclusion of treatment each practitioner (except those in the study group that had no patient questionnaires to distribute) passed on a survey kit to their sampled patient(s) containing a patient questionnaire, cover letter and explanation sheet. Sampled patients completing the patient questionnaire recorded basic socio-demographic characteristics and data concerning the nature, severity and duration of their symptoms. The primary rationale for this 2-stage sampling methodology was to allow linkage of dentist-assessed oral health status to patient perceptions of quality of life. The patient questionnaires were identified using the practitioner identification number allowing linkage between the practitioner logbook data and patient questionnaire data, but maintaining the anonymity of each patient to the investigators.

### Instrument development

A pilot study was conducted which collected five patients per dentist in order to establish the feasibility of the 2-stage methodology. Since the optimum number of patients to sample from dentists was not known, dentists in the main study were randomised into six equal-sized groups in order to assess the sample size-related efficiency and response properties of recording data on from 1 to 5 patients and distributing between 0 to 5 patient questionnaires.

### Sampling and data collection

A 2-stage sampling design was used where stage 1 involved sampling dentists and stage 2 involved sampling of patients within selected dentists. Dentists were randomly sampled from the South Australian Dental Register. The Dental Register was used as a sampling frame as it provides a comprehensive listing of all persons registered to work as dentists in the State of South Australia, and is therefore representative of the target population of dentists. Sampled dentists were randomised into one of seven equal-sized study groups to assess the optimum number of patients to sample from dentists and sent a mailed self-complete dentist questionnaire along with up to five self-complete patient questionnaires depending on the study group. Note that dentists in the group that had no patient questionnaires to distribute recorded details of 5 patients in their dentist questionnaire, while dentists in all other groups recorded the same number of patients in their dentist questionnaire as they distributed patient questionnaires.

Data were collected during 2001–02 with a primary approach letter sent initially to each dentist, followed a week later by the survey materials, with a reminder card two weeks later, and up to four follow-up mailings of survey materials to dentists who had not yet responded in order to ensure higher response rates [16].

The emphasis of the project was to obtain precise estimates of the component measures of the burden of oral disease. These are typically expressed as percentages, such as the percentage of persons or percentage of time experiencing symptoms of a given degree of severity. Taking a parameter size of 10% as a reference estimate for any given measure, in order to achieve a level of precision of 20% or less relative standard error, a minimum target sample of n = 225 patients was required. This would provide an acceptable level of precision for estimates as low as 10% in size, and better precision for any estimates larger than 10% in size.

### Data items

Dentists recorded the details of the dental conditions that patients had, and patients recorded their experience of those dental conditions. In the patient questionnaire, patients were asked if the dental conditions had caused problems in each of six health state dimensions using the European Quality of Life indicator or EuroQol (EQ-5D+) instrument [8]. The six health state dimensions were: mobility (e.g, walking about), self-care (e.g, washing, dressing), usual activities (e.g., work, study, housework, family or leisure), pain/discomfort, anxiety/depression and cognition (e.g, memory, concentration, coherence, IQ). The EuroQol measures each of these six dimensions according to a 3-level response grading from 1 = no problems, 2 = some/moderate problems and 3 = extreme problems. Patients were also asked to rate their experience of dental problems in the last year using the OHIP-14 [7], which uses 14 items to capture measures of the seven dimensions of functional limitation, physical pain, psychological discomfort, physical disability, psychological disability, social disability and handicap. For each of the 14 OHIP questions subjects were asked how frequently they had experienced impact in the preceding 12 months using a Likert-type response scale re-coded as a Guttman scale 4 = very often, 3 = fairly often, 2 = occasionally, 1 = hardly ever and 0 = never.

### Measures

The main output measure was calculated by converting EuroQol item responses to health state values, where each set of responses on the standard 5-item instrument was matched to a health state value where 0 = death and 1.0 = perfect health by an algorithm derived from modelling values [17] using health state preferences elicited from a general population [8]. The responses to the OHIP-14 instrument were coded into categories of 'Never', 'Hardly ever' and 'Occasionally/Fairly often/Very often' with each category converted into indicator variables with values of one if there was a response in that category or with values of zero if there was no response in that category. Age was entered in years and sex was coded as one for males and zero for females.

### Data analysis

**Figure 1:**
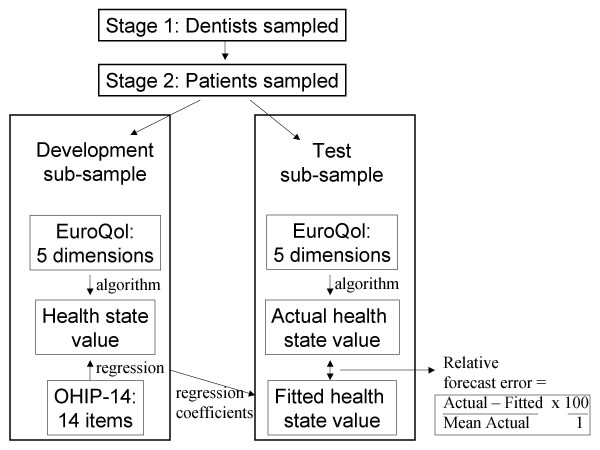
Outline of sampling and analysis.

The characteristics of responding patients were compared descriptively with published data on dental patients and the Australian population. A random sample of two thirds of the respondents was used to construct models of health state values (see Figure 1 for an outline of the sampling and analysis). Two tobit regression models were constructed using the EQ-5D based health state value as the dependent variable with the independent variables of OHIP-14 items, sex and age. Tobit regression was used to account for censoring of the characteristically bounded nature of health state values that can result in biased and inconsistent estimates using ordinary least-squares regression [18-20]. One model used the OHIP-14 items as categorical variables with the category 'Never' as the reference category, while the other model used the OHIP-14 item responses as continuous variables that were coded from 0 to 4. Cases with missing data on the dependent or independent variables were excluded from the analysis. Non-significant terms were retained for comparability across models, and their potential value in controlling for confounding [21]. The remaining one third sample of the respondents was used to test the models by comparing fitted versus actual values using forecast errors. Forecast errors were calculated by subtracting fitted values from actual health state values and dividing by the mean actual health state value to convert absolute forecast errors into relative forecast errors as a percentage of the actual sample mean health state value. Model building strategies also included testing model fit after inclusion of additional terms such as age-squared to model non-constant age effects and interactions between OHIP-14 items, and examining correlations of independent variables for possible collinearity. The design effect of clustering of patients within sampled dentists was calculated and used as a weight to adjust the reported confidence intervals.

## Results

### Response

A total of 378 dentists responded to the survey (response rate = 60%). Response rates between study groups varied from 49 to 70% and tended to be higher in study groups that required dentists to sample less patients, but the effect was not monotonic (Table 1). Data were available for 375 patients from the patient questionnaire, comprising a response rate of 72% of patients sampled, with response rates between study groups varying from 69 to 92%.

### Characteristics of patients

The characteristics of patients are presented in Table 2 where data from private general practice [22] and Australian population estimates [23,24] are presented for comparison. The majority of patients were female (59.5%), born in Australia (75.5%), had dental insurance (64.8%) and had visited a dentist in the last 12 months (65.3%). The main reason for dental visiting was for other dental problems not involving relief of pain (46.7%), followed by check-ups (35.2%) and emergency visits involving relief of pain (18.1%).

### Distribution of variables

The mean health state value was 0.852 (95% confidence interval 0.840 to 0.964). Table 3 shows that a minority of patients reported symptoms in the 'Occasionally/Fairly often/Very often' category, ranging from 3.6% for 'unable to function' to 46.0% for 'uncomfortable eating'. Responses in the 'hardly ever' category ranged from 6.9% for 'unable to function' to 29.0% for 'painful aching in the mouth'. Mean values for the OHIP-14 items ranged from 0.140 for 'unable to function' up to 1.355 for 'uncomfortable eating'.

### Regression models

The OHIP-14 items and sociodemographic covariates had relatively high pseudo R-squared values (Table 4). OHIP items relating to 'painful aching in mouth' were significantly related to health state values in both models while 'life less satisfying' was also significant in the continuous model. In each case, the regression coefficients were negative indicating that responses to the OHIP-14 items were associated with lower health state values.

Table 5 shows the mean forecast errors for both the categorical and continuous variable models. Mean forecast errors relative to the mean observed health state value were higher when fitted to the categorical model (17.4%) compared to the continuous model (15.2%), (P < 0.05). However, the forecast errors were not consistently higher for the categorical model at each level of observed health state value.

Table 6 demonstrates the relationship between observed and fitted values within selected ranges of observed health state values using the categorical model. At each level of observed health state values the fitted values slightly overestimated the observed health state value.

Note that alternative versions of the models were attempted using an age-squared term and also exploring interactions between OHIP items, but as they did not substantially alter pseudo R-squared values or forecast errors, the results for these additional models are not presented. Examination of correlations among the independent variables indicated that OHIP items were positively correlated with rho ranging between 0.19 and 0.84. The only correlation above 0.75 occurred between the items 'Felt self-conscious' and 'Been embarrassed'. However dropping one of these items had no effect on the direction of associations and only minimal effect on the pseudo R-squared value or the significance and magnitude of the regression coefficient, hence the full set of items were retained in the analysis.

## Discussion

### Response and representativeness

Response rates to the survey were adequate for both the dentist and patient questionnaires [25]. Comparison of respondents against estimates for private general practice and the Australian population indicated a slightly higher percentage of female patients compared to the population consistent with higher reported visiting rates by females [24], but both place of birth and time since last visit was similar. While dental insurance was higher, the percentage of check-up visits was lower among patients indicating a higher percentage of dental problems for patients compared to the population.

The use of data from a self-selected typical day was used to provide representative estimates. A report has shown that there was no significant difference in service rates in all 10 main areas of service between data collected over a 10-day sampling period compared with estimates based on one typical day nominated from the 10-day sampling period by the responding dentists [22].

### Instruments

Previous analysis of the EuroQol and OHIP-14 instruments found that both the generic and specific measures showed evidence of discriminant validity in detecting associations with visit characteristics and main dental condition being treated among dental patients. There was little difference by type of measure used with simple counts, additive scores and scale scores demonstrating discriminative ability in both bivariate and multivariate analyses [26]. The generic and specific instruments showed a degree of overlap in dimensions, particularly for pain [27]. The partial separation in the domains of both instruments confirms that generic and specific measures can be used in combination to capture different elements of quality of life – with both instruments covering symptom experience of pain but EuroQol tapping daily activities such as self-care and usual activities and OHIP tapping oral health-specific aspects of oral functional limitation and physical disability as well as psychological and social aspects of disability and handicap. There are, however, plausible potential links between the two descriptive systems. For example, mobility could in some persons be influenced by oral health problems, such as severe toothache that results in their seeking bed rest or limiting their movements. It is also worth noting that in this study the EQ-5D was asked in relation to dental problems, hence potential effects of co-morbidity on health state value would not be confounding the relationship between EQ-5D health states and OHIP-14 items.

As reported for population surveys [10], the distribution of the EuroQol was skewed among dental patients with a minority reporting problems on any one dimension [26]. The effect of skewness is to produce a ceiling effect where most of the responses are clustered at one extreme [28]. This ceiling effect was less marked for the pain/discomfort and anxiety/depression dimensions, but the large numbers of respondents reporting no problems may make the instrument less appropriate for studies of milder conditions [2]. Despite the simplicity of the EuroQol in terms of dimensions and response categories, there is growing evidence of its construct validity [10]. Similarly, the OHIP has also been noted as displaying ceiling effects even among dental patients [29], indicating limitations in both the generic and specific instruments as descriptive measures. Floor effects are just like ceiling effects but they are found at the opposite end of the scale. Floor effects, with high percentages reporting problems, have not been reported to be of the same extent for the EuroQol compared to other generic measures such as SF-6D or HUI3 making it more suitable for generating preference-based index values for use in economic evaluation when the conditions studied are more severe [2,30].

### Health state value algorithm

The present study provides an algorithm that transforms OHIP-14 scores into estimates of health state values. While both the models performed similarly, the lower forecast errors for the continuous model indicate that this may be preferred over the categorical model. This algorithm can then be used in QALY or DALY analyses from databases that contain OHIP-14 scores, but not health state values. While, the preferred method would be to derive health state valuations from a population sample, the mapping algorithm facilitates cost-effectiveness and burden of disease studies through proxy health state values that can be derived from the numerous oral health studies that have collected data using the OHIP instrument.

The proportion of variance explained by the algorithm from a previously reported mapping of a profile measure on an index was between 35% – 55% of the variance in HUI3 explained using SF-12 items [3]. In this study the values of pseudo R-squared were higher. However, values of pseudo R-squared are based on likelihood statistics from a model containing the independent variables versus a model containing a constant term only, rather than a comparison of fitted to observed values as obtained from linear regression models (31). Previous mapping studies have cautioned that the use of such mapped utility values would not be appropriate for use at the individual level and instead should be applied to analyses performed at the group level [3]. In the present study, the fitted mean was higher than the observed mean for the group of patients as a whole, and when stratified into different levels of health state value. Hence, an individual's predicted health state value may be an over- or under-estimate of the true health state if it were observed (i.e., directly measured), and as a group, health states would be slightly over-estimated. Such variation may be acceptable at a group level where aggregate health state values are of interest. However, some caution should be applied in interpreting findings as health states will tend to be slightly over-estimated (i.e., healthier than if directly measured) and in the case of disability weights for burden of disease studies the over-estimation of health states equates to lower or conservative estimates of disability weights (i.e., less disability than if directly measured). As participants in this study were dental patients who may have more oral disease than the general population the over-estimation of health states may be exacerbated when applied to general populations. The consistency of the over-estimation of health state values suggests that no systematic variation or bias would result from the application of the algorithm to sub-groups with different underlying health states.

## Conclusion

The approach enables health state values to be derived from OHIP-14 scores for populations where utility has not or cannot be measured directly.

## Competing interests

The author(s) declare that they have no competing interests.

## Authors' contributions

DSB and AJS were chief investigators on the grants obtained to fund the study. DSB performed data collection, analysis and drafting of the manuscript. AJS participated in the design and coordination of the study, and completion of the manuscript. All authors read and approved the manuscript.

## Pre-publication history

The pre-publication history for this paper can be accessed here:



## Figures and Tables

**Table 1:** Response to the dentist and patient questionnaires

		**Dentist questionnaire**				**Patient questionnaire**		
			
				**Patients recorded**		**Patients recorded**		
						
	**Patients sampled per dentist**	**Number of dentists responding**	**Response rate (%)**	**Number**	**Percent**	**Number**	**Percent**	**Response rate (%)**
**Pilot study**	5	60	(65)	135	(17.9)	93	(24.8)	(69)
**Main study (a)**	0	61	(70)	237	(31.4)	-	(-)	(-)
**Main study (b)**	1	56	(62)	37	(4.9)	29	(7.7)	(78)
**Main study (c)**	2	54	(60)	49	(6.5)	45	(12.0)	(92)
**Main study (d)**	3	43	(49)	61	(8.1)	41	(10.9)	(67)
**Main study (e)**	4	50	(58)	118	(15.6)	84	(22.4)	(71)
**Main study (f)**	5	54	(57)	119	(15.7)	83	(22.1)	(70)

**Total**		**378**	**(60)**	**756**	**(100.0)**	**375**	**(100.0)**	**(72)**

**Table 2:** Characteristics of patients compared with private general practice and Australian population estimates

	**Burden of Oral Disease Study (n = 375)**	**Private general practice^(a) ^** **(n = 4,858)**	**Australian population** **(n = 7,829)**
	% [95% CI]	%	%
**Sex**			
% Female	59.5 [54.3–64.7]	54.9	^(b) ^50.4
**Place of birth**			
% Australian	75.5 [70.9–80.1]	n.a.	^(b) ^76.4
**Dental insurance status**			
% Insured	64.8 [59.7–69.9]	47.8	^(c) ^34.8
**Reason for dental visit**			
Check-up	35.2 [30.1–40.3]	41.1	^(c) ^45.1
Emergency	18.1 [16.0–20.2]	28.6	n.a.
Other dental problem	46.7 [41.4–52.0]	30.8	n.a.
**Time since last dental visit**			
% visited in last 12 months	65.3 [60.2–70.4]	n.a.	^(c) ^61.3

(a): Longitudinal Study of Dentists' Practice Activity 1998–99

(b): Australian Bureau of Statistics 2002

(c): National Dental Telephone Interview Survey 1999

n.a.: denotes data not available

**Table 3:** Descriptive statistics for OHIP-14 items as categorical and continuous variables

				**95% CI**	
				
**Variable**	**n**	**Percent**	**Mean**	**Lower bound**	**Upper bound**
**Trouble pronouncing words**	365		0.296	0.223	0.369
Never		81.6			
Hardly ever		10.1			
Occas., F/Often, V/Often		8.2			
**Sense of taste worsened**	356		0.399	0.315	0.483
Never		76.1			
Hardly ever		11.5			
Occas., F/Often, V/Often		12.4			
**Painful aching in mouth**	359		1.100	0.990	1.211
Never		36.5			
Hardly ever		29.0			
Occas., F/Often, V/Often		34.5			
**Uncomfortable eating**	361		1.355	1.230	1.479
Never		32.4			
Hardly ever		21.6			
Occas., F/Often, V/Often		46.0			
**Felt self-conscious**	361		1.094	0.961	1.227
Never		48.8			
Hardly ever		15.0			
Occas., F/Often, V/Often		36.3			
**Felt tense**	362		0.820	0.705	0.936
Never		57.2			
Hardly ever		16.3			
Occas., F/Often, V/Often		26.5			
**Diet unsatisfactory**	359		0.457	0.365	0.549
Never		73.8			
Hardly ever		12.5			
Occas., F/Often, V/Often		13.7			
**Interrupt meals**	363		0.548	0.460	0.636
Never		64.2			
Hardly ever		20.7			
Occas., F/Often, V/Often		15.2			
**Difficulty relaxing**	362		0.597	0.499	0.695
Never		64.9			
Hardly ever		16.9			
Occas., F/Often, V/Often		18.2			
**Been embarrassed**	365		0.800	0.686	0.914
Never		57.0			
Hardly ever		18.6			
Occas., F/Often, V/Often		24.4			
**Irritable with other people**	364		0.525	0.436	0.613
Never		66.5			
Hardly ever		18.7			
Occas., F/Often, V/Often		14.8			
**Difficulty doing usual jobs**	365		0.282	0.216	0.348
Never		80.3			
Hardly ever		12.6			
Occas., F/Often, V/Often		7.1			
**Life less satisfying**	363		0.562	0.464	0.660
Never		68.6			
Hardly ever		13.2			
Occas., F/Often, V/Often		18.2			
**Unable to function**	363		0.140	0.095	0.186
Never		89.5			
Hardly ever		6.9			
Occas., F/Often, V/Often		3.6			

Note: "Occas., F/Often, V/Often" refers to "Occasionally, Fairly often, Very often"

**Table 4:** Regression models of Health state values by OHIP-14

	**Categorical model^(1) ^(n = 209)**			**Item model (n = 209)**		
		
**Variable**	**Coef.**	**S.E.**	**P**	**Coef.**	**S.E.**	**P**
**Trouble pronouncing words**				0.0154	(.025)	0.544
Hardly ever	0.0826	(.054)	0.128			
Occas., F/Often, V/Often	0.0750	(.072)	0.296			
**Sense of taste worsened**				0.0020	(.022)	0.930
Hardly ever	-0.3670	(.062)	0.557			
Occas., F/Often, V/Often	-0.0238	(.056)	0.674			
**Painful aching in mouth**				-0.0905	(.020)	0.000
Hardly ever	-0.1192	(.047)	0.011			
Occas., F/Often, V/Often	-0.2108	(.054)	0.000			
**Uncomfortable eating**				-0.0173	(.019)	0.362
Hardly ever	-0.0154	(.052)	0769			
Occas., F/Often, V/Often	-0.0405	(.052)	0.442			
**Felt self-conscious**				0.0281	(.026)	0.273
Hardly ever	-0.0830	(.050)	0.096			
Occas., F/Often, V/Often	0.0375	(.064)	0.559			
**Felt tense**				0.0289	(.027)	0.282
Hardly ever	0.0893	(.053)	0.097			
Occas., F/Often, V/Often	0.0547	(.062)	0.377			
**Diet unsatisfactory**				0.0032	(.024)	0.894
Hardly ever	-0.0155	(.057)	0.787			
Occas., F/Often, V/Often	-0.0595	(.060)	0.320			
**Interrupt meals**				-0.0337	(.025)	0.188
Hardly ever	-0.0074	(.049)	0.880			
Occas., F/Often, V/Often	-0.0483	(.064)	0.448			
**Difficulty relaxing**				-0.0187	(.030)	0.531
Hardly ever	-0.0770	(.057)	0.176			
Occas., F/Often, V/Often	-0.1074	(.066)	0.106			
**Been embarrassed**				0.0055	(.029)	0.850
Hardly ever	-0.0115	(.052)	0.825			
Occas., F/Often, V/Often	0.0462	(.067)	0.489			
**Irritable with other people**				-0.0186	(.030)	0.535
Hardly ever	0.0598	(.054)	0.266			
Occas., F/Often, V/Often	-0.0526	(.066)	0.429			
**Difficulty doing usual jobs**				-0.0266	(.038)	0.481
Hardly ever	-0.0745	(.059)	0.207			
Occas., F/Often, V/Often	-0.0113	(.096)	0.906			
**Life less satisfying**				-0.0550	(.026)	0.034
Hardly ever	-0.0502	(.057)	0.382			
Occas., F/Often, V/Often	-0.0646	(.063)	0.307			
**Unable to function**				-0.0177	(.037)	0.633
Hardly ever	-0.0387	(.080)	0.631			
Occas., F/Often, V/Often	-0.0837	(.084)	0.319			
**Sex (male)**	0.0375	(.034)	0.271	0.0204	(.031)	0.518
**Age (years)**	-0.0005	(.001)	0.686	0.0002	(.001)	0.833
**Intercept**	1.1466	(.071)	0.000	1.0822	(.060)	0.000
						
**Model:**			0.0000			0.0000
**Pseudo R-squared**	0.66			0.62		

(1): Reference category: Never

Note: "Occas., F/Often, V/Often" refers to "Occasionally, Fairly often, Very often"

**Table 5:** Mean relative forecast errors within categories of observed health state values for categorical and item models

		**Categorical model**		**Item model**	
			
**Observed health state value category**	**n**	**Mean**	**[95% CI]**	**Mean**	**[95% CI]**
<0.70	7	28.7%	[8.2–49.1]	24.6%	[10.8–38.4]
0.70–<0.80	10	13.2%	[6.0–20.3]	17.3%	[8.6–26.0]
>0.80	97	17.1%	[14.9–19.2]	14.4%	[12.6–16.1]
					
All	114	17.4%	[15.2–19.6]	15.2%	[13.5–17.0]

**Table 6:** Mean observed and fitted health state values within categories of observed health state values: categorical model

		**Observed values**		**Predicted values**	
			
**Observed health state value category**	**n**	**Mean**	**[95% CI]**	**Mean**	**[95% CI]**
<0.70	7	0.50	[0.38–0.61]	0.61	[0.42–0.82]
0.70–<0.80	10	0.73	[0.72–0.75]	0.78	[0.68–0.88]
>0.80	97	0.89	[0.88–0.90]	0.95	[0.92–0.99]
					
All	114	0.85	[0.83–0.87]	0.91	[0.88–0.95]

## References

[B1] Allen PF (2003). Assessment of oral health related quality of life. Health Qual Life Outcomes.

[B2] Brazier JE, Roberts J (2004). The estimation of a preference-based measure of health from the SF-12. Med Care.

[B3] Sengupta N, Nichol MB, Wu J, Globe D (2004). Mapping the SF-12 to the HUI3 and VAS in a managed care population. Med Care.

[B4] Brennan DS, Spencer AJ (2004). Disability weights for the burden of oral disease in South Australia. Pop Health Metrics.

[B5] Slade GD, Spencer AJ (1994). Development and evaluation of the oral health impact profile. Community Dent Health.

[B6] Locker D (1988). Measuring oral health: a conceptual framework. Community Dent Health.

[B7] Slade GD (1997). Derivation and validation of a short-form oral health impact profile. Community Dent Oral Epidemiol.

[B8] Brooks R (1996). EuroQol: the current state of play. Health Policy.

[B9] Bowling A (2001). Measuring disease A review of disease-specific quality of life measurement scales.

[B10] Johnson JA, Coons SJ (1998). Comparison of the EQ-5D and SF-12 in an adult US sample. Qual Life Res.

[B11] Kind P, Dolan P, Gudex C, Williams A (1998). Variations in population health status: results from a United Kingdom national questionnaire survey. BMJ.

[B12] Stouthard MEA, Essink-Bot M-L, Bonsel GJ (2000). Disability weights for diseases. A modified protocol and results for a Western European region. European J Public Health.

[B13] Anand S, Hanson K (1997). Disability-adjusted life years: a critical review. J of Health Economics.

[B14] Murray CJL, Lopez AD, Jamison DT (1994). The global burden of disease in 1990: summary results, sensitivity analysis and future directions. Bull World Health Organ.

[B15] Mathers C, Vos T, Stevenson C (1999). The burden of disease and injury in Australia.

[B16] Dillman DA (1978). Mail and telephone surveys The total design method.

[B17] Dolan P (1997). Modeling valuations for EuroQol health states. Med Care.

[B18] Austin PC, Escobar M, Kopec JA (2000). The use of the Tobit model for analysing measures of health status. Qual Life Res.

[B19] Green WH (2000). Econometric analysis.

[B20] Maddala GS (1999). Limited-dependent and qualitative variables in econometrics.

[B21] Rothman KJ (1986). Modern epidemiology.

[B22] Brennan DS, Spencer AJ (2002). Dentists' practice activity in Australia: 1983–84 to 1998–99.

[B23] Australian Bureau of Statistics (2002). Australian demographic statistics – March quarter 2002.

[B24] Carter KD, Stewart JF, Spencer AJ (2001). National dental telephone interview survey 1999.

[B25] Mangione TW (1995). Mail surveys Improving the quality.

[B26] Brennan DS, Spencer AJ (2005). Comparison of a generic and a specific measure of oral health related quality of life. Community Dent Health.

[B27] Brennan DS, Spencer AJ (2004). Dimensions of oral health related quality of life measured by the EQ-5D+ and OHIP-14. Health Qual Life Outcomes.

[B28] Streiner DL, Norman GR (1995). Health measurement scales A practical guide to their development and use.

[B29] Locker D, Matear D, Stephens M, Lawrence H, Payne B (2001). Comparison of the GOHAI and OHIP-14 as measures of the oral health-related quality of life of the elderly. Community Dent Oral Epidemiol.

[B30] Brazier JE, Harper R, Munro J, Walters SJ, Snaith ML (1999). Generic and condition-specific outcome measures for people with osteoarthritis of the knee. Rheumatology.

[B31] Selvin S (1996). Statistical analysis of epidemiologic data.

